# Priming and bonding metal, ceramic and polycarbonate brackets

**DOI:** 10.1080/26415275.2019.1684823

**Published:** 2019-11-06

**Authors:** Leeni Kilponen, Juha Varrela, Pekka K. Vallittu

**Affiliations:** a Institute of Dentistry, University of Turku, Turku, Finland;; b Division of Welfare, Turku, Finland;; c Department of Oral Development and Orthodontics, University of Turku, Turku, Finland;; d Department of Biomaterials Science, Turku Clinical Biomaterials Centre – TCBC, Turku, Finland

**Keywords:** Orthodontic brackets, dental bonding, primers, bond strength, enamel damage

## Abstract

**Objective:** To investigate if primers can be used to modify bonding characteristics of orthodontic brackets.

**Materials and methods:** Stainless steel, zirconia-alumina ceramic and polycarbonate brackets were bonded to enamel with and without universal and bracket material specific primers on the bracket base. Orthodontic adhesive cement (Transbond™XT) was used for bonding. The primers in each group (*n* = 10) were silane based (RelyX™ Ceramic Primer) and universal primer (Monobond Plus) for ceramic and metal brackets, and adhesive resin (Adper™ Scotchbond™ Multi-Purpose Adhesive) and composite primer (GC Composite Primer) for polycarbonate brackets. Controls with no primer were used for all bracket types. Teeth with bonded brackets were stored in distilled water in 37 °C for 7 days and debonded with static shear loading. Debonding forces were recorded and analyzed with ANOVA. Adhesive remnant index (ARI) was determined and enamel damage examined.

**Results:** The bond strength without primers was 8.14 MPa (±1.49) for metal, 21.9 MPa (±3.55) for ceramic and 10.47 MPa (±2.11) for polycarbonate brackets (*p* < .05). Using silane as primer increased the bond strength of ceramic brackets significantly to 26.45 MPa (±5.00) (*p* < .05). ARI-scores were mostly 2–3 (>50% of the adhesive left on the enamel after debonding), except with silane and ceramic brackets, ARI-score was mostly 0–1 (>50% of the adhesive left on the bracket). Debonding caused fractured enamel in four specimens with ceramic brackets.

**Conclusions:** Bond strength was highest for ceramic brackets. Silane primer increased bond strength when used with ceramic brackets leading to enamel fractures, but otherwise primers had only minor effect on the bond strength values.

## Graphical Abstract

**Figure:**
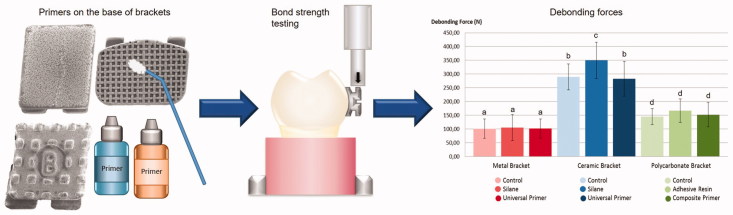

## Introduction

In fixed orthodontic appliances, the bracket-enamel adhesion should provide a strong attachment of the bracket during treatment, but allow debonding of the bracket without enamel damage at the end of the treatment. The rate of bracket failure is found to be varied, but somewhere between 2-20% of the brackets fail prematurely during the treatment [1–3]. The bond strength can be modified by affecting the properties of the adhesive cement or by increasing the mechanical retention by changing the design of the bracket base. In case of bonding failure or in post-treatment removal of the brackets, the break-off can take place either between the bracket and the adhesive cement or between the adhesive cement and the enamel, or at both interfaces. A too high bonding strength may lead to breakage inside enamel or even at the dentino-enamel junction when the bracket is removed [4].

There are many different types of brackets available, the most common materials being metal, ceramic and polycarbonate. The brackets are bonded to enamel with light-curing adhesive cement. When bonding metal brackets, penetration of light under the bracket is limited, and without chemical bonding between the metal bracket and the adhesive cement, the bond strength tends to remain low compared to translucent or chemically bondable ceramic brackets [5–7]. Consequently, with metal brackets there is a higher risk for bracket failure but on the other hand, removing brackets is easy. A break-off usually takes place at the bracket-adhesive interface, and enamel damage is only rarely encountered [8–11]. The bond strength of metal brackets can be improved e.g. by sandblasting, microetching and silanation of the bracket base [12–15].

Translucent ceramic brackets allow a more complete photopolymerization of the adhesive, and some ceramic brackets rely on chemical bonding in addition to mechanical retention, resulting in high bond strength [4,7,9,16–18], but because of the strong attachment, there is a higher risk for enamel damage during bracket removal. Similarly to ceramic brackets, polycarbonate brackets are translucent but they are reported to have lower bonding properties than ceramic or metal brackets [19,20].

Primers are used in dentistry to promote adhesion between dissimilar substrates that do not naturally bond with each other. Primers are substrate specific, and with some substrates, chemical bonding can be achieved. However, despite of their surface specificity, improvement of wettability of the bonding surface by the primer is a common property of all primers. Silane based primers are used with ceramic and also with metal substrates, but for polymer composite substrates there are specific primers. Recently universal primers, which can be used with various types of substrates, have been introduced [21,22].

The objective of this study was to investigate whether different primers could be used to modify the bonding characteristics of metal, ceramic and polycarbonate brackets to achieve adequate bond strength without increasing the risk of enamel damage at debonding.

## Materials and methods

Brackets of three different materials (stainless steel, zirconia-alumina ceramic and polycarbonate) were bonded to enamel using bracket material specific or universal primers on the bracket base with orthodontic adhesive cement (Transbond™XT). The brackets were upper central incisor brackets, Inspire ICE by Ormco (a ceramic monocrystalline aluminum oxide bracket with a base covered in small zirconia spheres), Spirit MB by Ormco (a filler reinforced polycarbonate bracket), and Ortomat Minimat by Ormco (a stainless steel bracket). The brackets of each material were divided in three groups (*n* = 10) according to the primer used in the bonding procedure. The primers were selected to match the different bracket types based on their universal affinity or material specificity for the bracket materials, and they were a silane based primer (RelyX™ Ceramic Primer) and a universal primer (Monobond Plus) for ceramic and metal brackets, and adhesive resin (Adper™ Scotchbond™ Multi-Purpose Adhesive) and a composite primer (GC Composite Primer) for polycarbonate brackets. Two types of adhesion promoters, methacryloxypropyltrimethoxysilane (MPS) of the silane based primer, and methacrylated phosphoric acid ester (MDP) combined with MPS of the universal primer, were chosen to be used with ceramic and metal brackets because of their ability to bond with multiple types of substrates. For the polycarbonate brackets, composite primer is specifically aimed at bonding between composite substrates, and the adhesive resin was chosen because of a similar solubility parameter between polycarbonate and BIS-GMA, which would allow the primer to dissolve and penetrate into the polycarbonate. A control group with no primer was used with all bracket types. The brackets and primers used in the study are listed in Table 1.

**Table 1.** Materials used in the study.

Materials	Manufacturer	Lot No.	Contents	Wt%
Transbond XT Light Cure Adhesive	3M Unitek (Monrovia, CA, USA)	N765918	Silane treated quartz BIS-GMA EBPADMA Silane treated silica Diphnyliodonium hexafluorophosphate	70–80* 10–20* 5–10* <2* <0.2*
Transbond XT Primer	3M Unitek (Monrovia, CA, USA)	N762529	BIS-GMA TEGDMA Triphenylantimony 4-(dimethylamino)-benzeneethanol DL-camphorquinone Hydroquinone	45–55* 45–55* <1* <0.5* <0.3* <0.03*
Etching Gel	3M Unitek (Monrovia, CA, USA)	626002	Water Phosphoric acid Amorphous silica	55–65* 30–40* 5–10*
RelyX™ Ceramic Primer	3M™ ESPE™, (St. Paul, MN, USA)	N759704	Ethyl alcohol Water Methacryloxypropyltrimethoxysilane	70–80* 20–30* <2*
Monobond Plus	Ivoclar Vivadent, (Schaan, Liechtenstein)	V12120	Ethanol 3-trimethoxysilylpropylmethacrylate Methacrylated phosphoric acid ester	50–100 ≤2.5 ≤2.5
Adper™ Scotchbond™ Multi-Purpose Adhesive	3M™ ESPE™, (St. Paul, MN, USA)	N735458	BIS-GMA 2-hydroxyethyl methacrylate (HEMA) Triphenylantimony	60–70* 30–40* <0.5*
GC Composite Primer	GC, (Hongo, Bunkyo-ku, Tokyo, Japan)	1604061	2-hydroxyethyl methacrylate (HEMA) Tetrahydrofurfuryl methacrylate Urethane dimethacrylate (UDMA)	30–60* 10–30* 10–30*
Ortomat Minimat	Ormco, (Glendora, CA, USA)	15M378M	Stainless steel	
Inspire ICE	Ormco, (Glendora, CA, USA)	081650467	Single crystal aluminum oxide Zirconium oxide	
Spirit MB	Ormco, (Glendora, CA, USA)	081612367	Filler reinforced polycarbonate	

BIS-GMA indicates bisphenol-A-diglycidyl ether dimethacrylate, EBPADMA bisphenol-A-bis(2-hydroxyethyl ether) dimethacrylate, TEGDMA triethylene glycol dimethacrylate.

Methacryloxypropyltrimethoxysilane and 3-trimethoxysilylpropylmethacrylate are different names used by the manufacturers for the same compound.

* The specific chemical identity and/or exact percentage (concentration) of this composition has been withheld as a trade secret.

The teeth used in the study were extracted molars acquired from the teaching clinic of Institute of Dentistry, University of Turku, Turku, Finland. The teeth were examined and only sound molars with sufficiently large and not too curved enamel areas similar to upper central incisors were included. The teeth were embedded vertically to blocks of acrylic resin so that the roots were inside the acrylic, they were cleaned with pumice, etched for 15 s using a 32% phosphoric acid etching gel, rinsed and air-dried. The selected primer was applied on the base of the bracket and air-dried/light cured according to the manufacturer’s instructions (Table 2), Transbond XT primer was applied on enamel, a small amount of Transbond XT adhesive cement was applied on the bracket base and the bracket was placed firmly on the enamel. Excess adhesive cement was removed with an instrument and the adhesive cement was light cured for 10 s (5 s from both sides) according to the manufacturer’s instructions. The specimens were stored in distilled water in 37 °C for 7 days and debonded with static loading using a testing machine (LLOYD Instruments, AMETEK Lloyd Instruments Ltd, West Sussex UK) with so-called shear-bond strength test with cross-head speed of 1 mm/min. The tip of the testing blade was positioned above the bracket wings close to the bracket base, the distance of the tip from the bracket base varying between 0.5–1 mm due to the differences in the thickness of the brackets, the metal brackets being thinner than ceramic or polycarbonate brackets. Debonding force and load-displacement curve were recorded. Testing was made in air at room temperature. After the testing, the specimens were analyzed and adhesive remnant index (ARI) (Table 3) and enamel damage were determined using a stereomicroscope (Wild 3MZ stereomicroscope, Wild Heerbrugg, Geis, Switzerland). Statistical analysis was performed with SPSS Statistics version 22.0 using Kruskall–Wallis test. Few specimens (one specimen from met1, cer1 and polyc1 groups each) were not included in the results due to testing machine error.

**Table 2.** Test groups, primers and the primer applying procedure.

Bracket type	Group name	Primer (applied to the base of the bracket)	Primer applying procedure
Metal	met1	No Primer	–
	met2	Silane (RelyX™ Ceramic Primer)	Gentle air drying
	met3	Universal Primer (Monobond Plus)	Gentle air drying
Ceramic	cer1	No Primer	–
	cer2	Silane (RelyX™ Ceramic Primer)	Gentle air drying
	cer3	Universal Primer (Monobond Plus)	Gentle air drying
Polycarbonate	pol1	No Primer	–
	pol2	Adhesive Resin (Adper™ Scotchbond™ Multi-Purpose Adhesive)	Gentle air drying, 10s light curing
	pol3	Composite Primer (GC Composite Primer)	Gentle air drying, 20s light curing

**Table 3.** Adhesive remnant index (ARI), definition of scores.

Score	Definition
0	No adhesive remained on enamel
1	Less than 50% of adhesive remained on enamel
2	More than 50% of adhesive remained on enamel
3	All adhesive remained on enamel

The morphology of bracket bases and fractured enamel were imaged using a scanning electron microscope (SEM, JSM-5500, Jeol USA, Inc., Peabody, MA) and an optical non-contacting profiler (Contour-GT-K1, Bruker, Billerica, MA, USA). The specimens were gold-sputtered and imaged. The profile data were analyzed with Bruker Vision 64 software (version 5.41, update 4, Bruker, Billerica, MA, USA).

## Results

**Figure 1.**
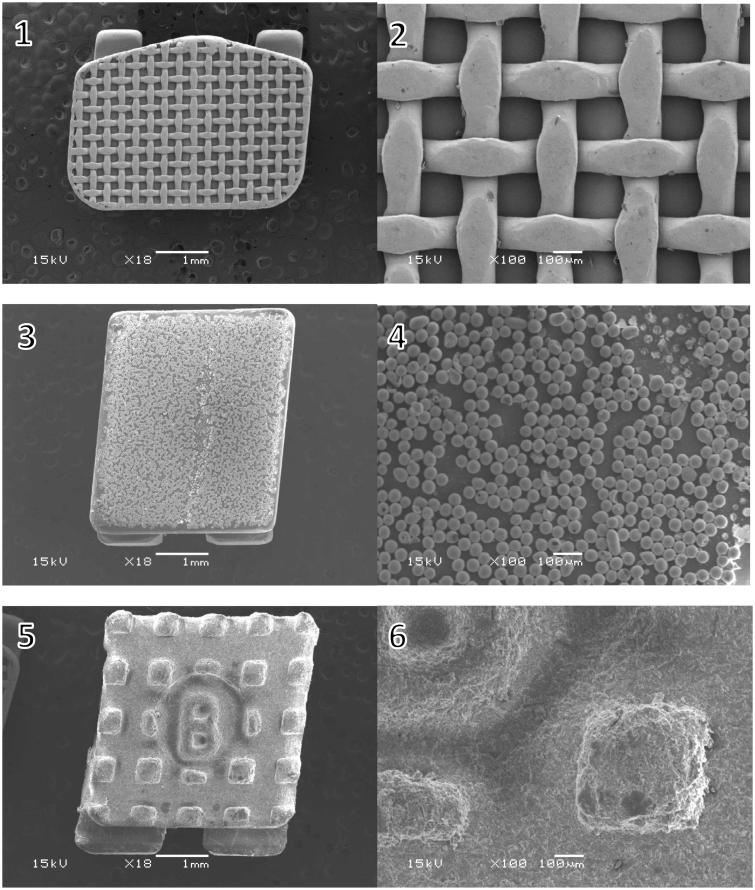
Scanning electron microscope images of the bracket bases. (1, 2) metal bracket with a mesh base design, magnification X18 and X100; (3, 4) ceramic bracket with small spheres on the base, magnification X18 and X100; (4, 5) polycarbonate bracket with large protuberances on the base, magnification X18 and X100.

**Figure 2.**
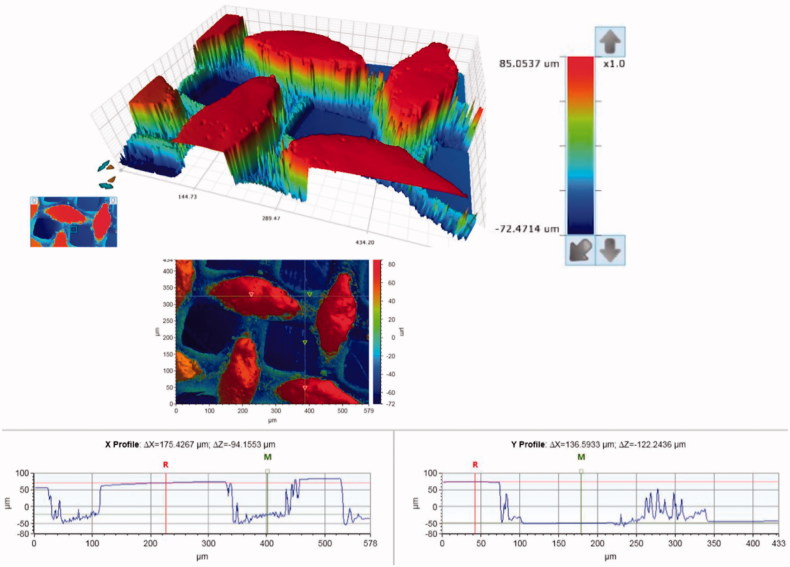
Surface contour analysis of the metal bracket, a close-up of the mesh on the bracket base. The overall difference between the highest and the lowest point of the base is approximately 125 µm.

**Figure 3.**
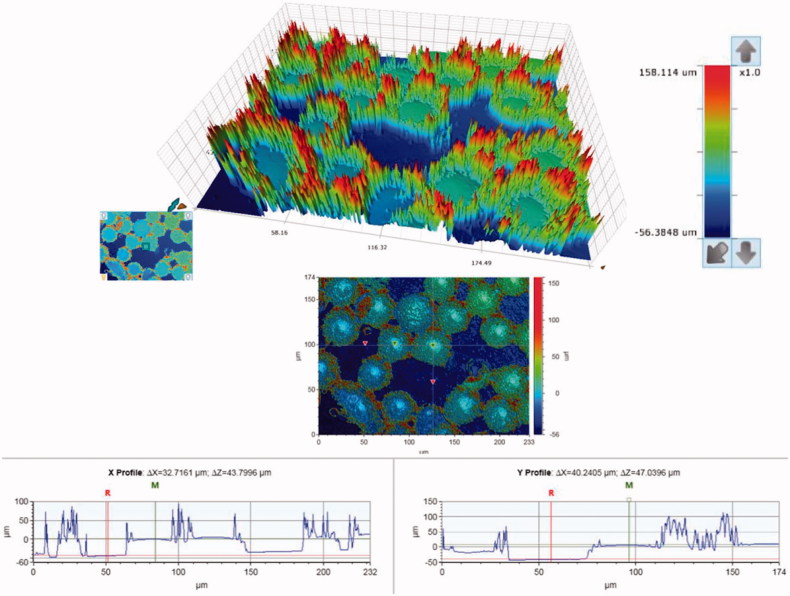
Surface contour analysis of the ceramic bracket, a close-up of the small spheres on the bracket base. The contour of the spheres accounts for the artifact around the edges of the spheres (the spikes). The overall difference between the highest and the lowest point of the base is approximately 50 µm.

**Figure 4.**
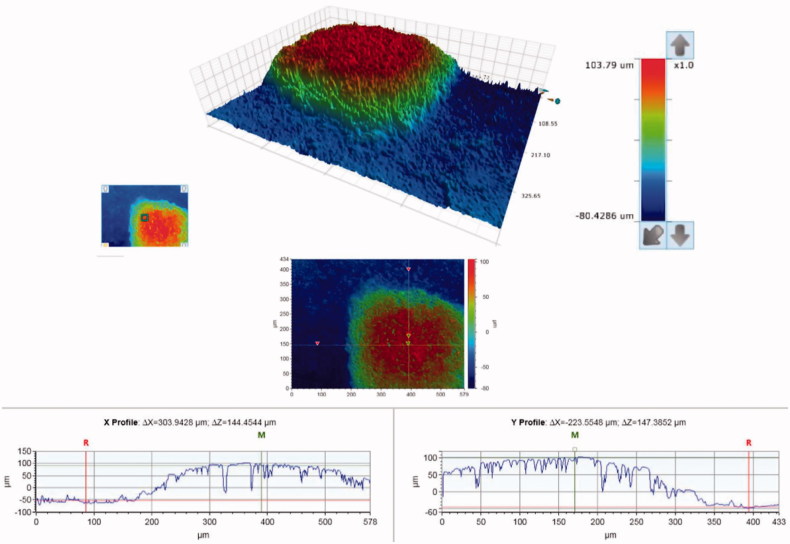
Surface contour analysis of the polycarbonate bracket, a close-up of one of the square protuberances on the bracket base. The overall difference between the highest and the lowest point of the base is approximately 150 µm.

SEM micrographs of the brackets are presented in Figure 1. The brackets had different base designs: the metal bracket had a mesh base, the ceramic bracket base was covered with small spheres (Ø approximately 40 µm) and the polycarbonate base had large square protuberances of varying sizes (approximately 200-500 µm). There was considerable variance in the height of the texture on the bracket bases between different bracket types: the difference between the highest and the deepest point in the base was approximately 125 µm for the metal, 50 µm for the ceramic, and 150 µm for the polycarbonate bracket, as can be seen in the profile graphs in Figures 2–4.

**Figure 5.**
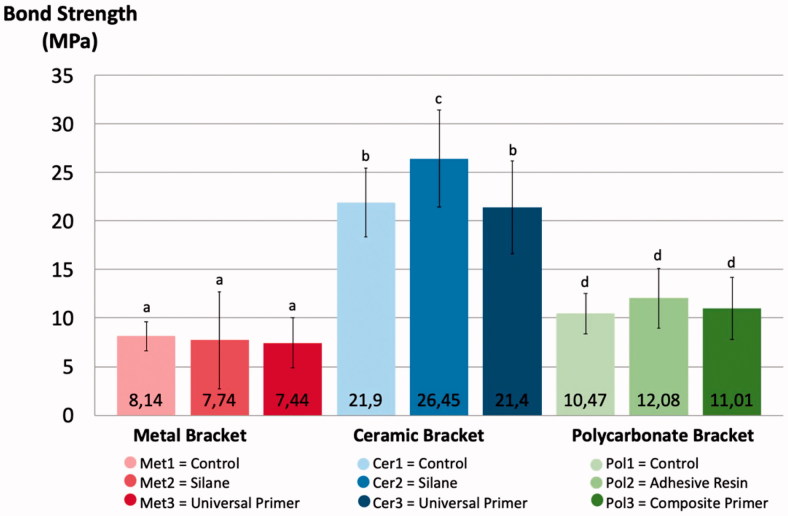
Bond Strength (MPa) of test groups, error bars represent standard deviation, the different letters above the columns represent statistically significant differences between groups (*p* < .05).

None of the brackets fractured during testing. The bond strength of brackets without primers was 8.14 MPa (±1.49) for metal, 21.9 MPa (±3.55) for ceramic and 10.47 MPa (±2.11) for polycarbonate brackets, all the values differed significantly (*p* < .05) (Figure 5). There were no differences between different primers and control group with metal or polycarbonate brackets. The bond strength of ceramic brackets used with silane primer was 26.45 MPa (±5.00), which was significantly higher compared to the control group and the universal primer group (*p* < .05).

**Figure 6.**
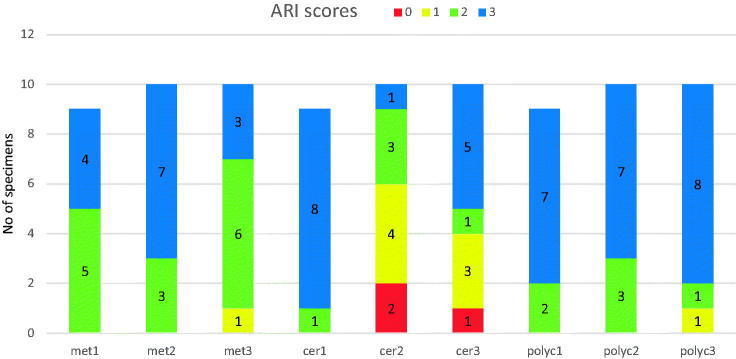
ARI scores of the test groups. See Table 3 for ARI score description.

**Figure 7.**
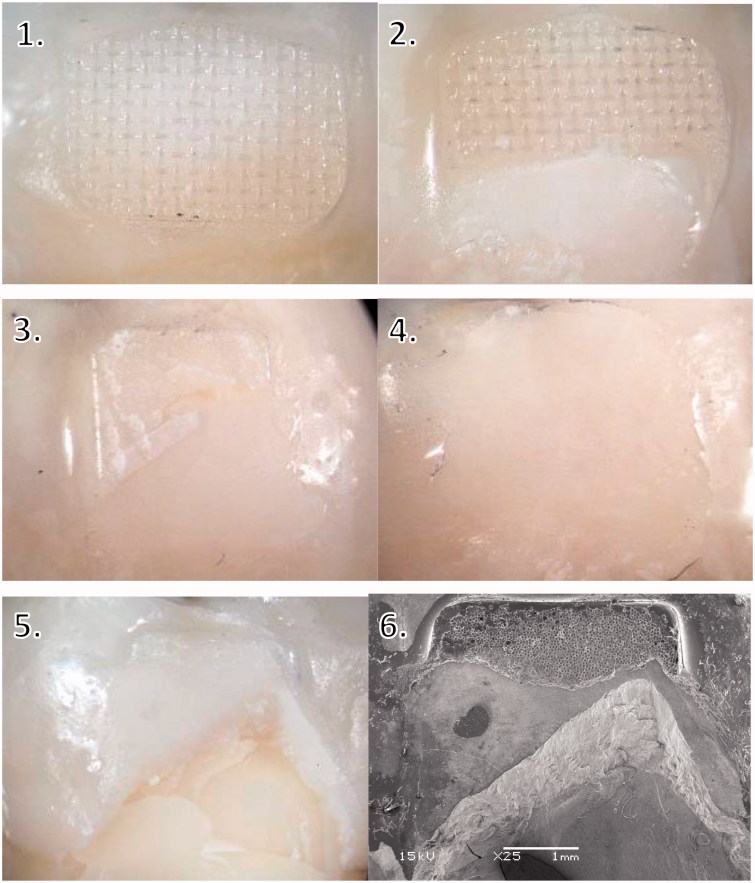
Examples of ARI scores. 1–5 light microscope images. 1 = ARI score 3, 2 = ARI score 2, 3 = ARI score 1, 4 = ARI score 0, 5 = ARI score 1 with enamel fracture, 6 = SEM image of the same sample as picture 5: adhesive remnants and fractured enamel, magnification X25.

ARI scores were mostly 2–3, except when silane primer was used with ceramic brackets, where the ARI score was mostly 0–1 (Figure 6). ARI scores 2–3 indicate that all or above 50% of the adhesive remained on the enamel after debonding, whereas ARI scores 0–1 indicate that all or above 50% of the adhesive remained on the bracket (Table 3). Stereomicroscope images of different ARI scores can be seen in Figure 7 (Images 1–4).

An enamel fracture was observed in four specimens using ceramic brackets: three with the silane based primer and one with the universal primer. No enamel fractures were observed with ceramic brackets without primer, or metal or polycarbonate brackets. As a group, ceramic brackets had significantly higher amount of enamel fractures compared to metal or polycarbonate brackets (*p* < .05). Stereomicroscope and SEM images of a specimen with fractured enamel can be seen in Figure 7 (Images 5–6).

## Discussion

Due to modern resin composites and the acid etching technique, the bond strength between enamel and the resin composite is quite high [23,24]. Therefore if the bond between the bracket and the adhesive cement also becomes very strong, the risk for enamel damage increases.

In this study, ceramic brackets yielded significantly higher bond strength than metal or polycarbonate brackets. Higher bond strength of ceramic brackets, especially with chemical bonding, can result in enamel damage during debonding [25,26], which was evident also in this study in group cer2 with silane primer, where enamel fractures were observed in three specimens (Figure 7, images 5–6). In fact, to avoid enamel fractures, bonding of ceramic brackets should be based on mechanical retention rather than on chemical bonding, as other studies have also suggested [16,18]. An additional risk for enamel damage is caused by the low fracture toughness of the ceramic brackets, especially the monocrystalline brackets, which may lead to break-ups of the bracket itself during debonding [17,27–32]. A remaining part of the ceramic bracket on enamel can be shaped in a way that it cannot be removed with pliers, and therefore needs to be removed with a rotary instrument. Due to the hardness of the ceramic bracket, it needs to be done with a diamond bur, but this can result in enamel damage [33]. It has been reported that the chance of enamel fracture during debonding of ceramic brackets could be diminished e.g. by using laser in the debonding procedure [34], or by applying the debonding force by compression rather than by shearing off the bracket, since this would lead to more favorable stress distribution in the enamel [35]. The compression from two sides of the bracket models the clinical case of using pliers, but it has been found that there is no difference in the failure mode between using pliers to detach brackets and shearing them off with a testing machine [20].

Because of the risk for enamel damage, it is usually considered safer if the breakage happens at the bracket-adhesive interface, even though higher ARI values leave more cement to be cleaned from the enamel surface [36]. The findings of the present study showed that when the bond strength between the bracket and the adhesive cement was not increased, the breakage usually happened at the bracket-adhesive interface, but stronger bonding, achieved by added chemical retention, resulted in lower ARI scores, i.e. breakage of the bond at the enamel-adhesive interface (Figures 6 and 7) or even inside enamel. Similar findings have been reported in earlier studies [13,37]. ARI score values of 0 and 1 were found significantly more often when primers were used with ceramic brackets (Figure 6). When ceramic brackets were bonded without primers, most adhesive cements was left on enamel, which is in accordance with the findings of previous studies [17,33,38]. The remaining adhesive cement must be removed, and a small damage to the enamel seems inevitable, but using e.g. a carbide bur for the clean-up is less destructive to enamel than removing a piece of a bracket with a diamond bur [39].

Silanes are alcohols containing a silicon (Si) atom. The most commonly used silane in dentistry is methacryloxypropyltrimethoxysilane (MPS), which contains a methacrylate group and three alkoxy-groups attached to a Si-atom. The methacrylate group reacts with the methacrylate groups in the composite resin, and the alkoxy groups are hydrolyzed and form acidic silanol groups, which then form bonds with the hydroxyl (-OH) groups on the surface of the substrate, e.g. glass ceramic [21,22,40]. When bonding to substrates that contain silica, which is spontaneously covered by -OH groups from the ambient moisture, siloxane linkages (-Si-O-Si-) are formed, and thus, the resin is covalently bound to the silica surface [41]. Weaker adhesion is achieved to metals (-Si-O-M-), because of fewer -OH groups on the oxidized metal surface. Silanes cannot sufficiently bond to chemically more inert substrates, e.g. oxide ceramics such as fully crystallized zirconia [42]. However, the surface of inert ceramic can be conditioned for chemical reactivity with silanes, e.g. chemical bonding to zirconia is possible with tribochemical silica-coating conditioning [43,44]. In addition, other adhesion promoters such as organophosphate ester monomers (MDP) can be used to enhance bonding to oxide ceramics [42,45–47]. It has been suggested that the bond strength of ceramic brackets could be further increased by mechanical retention, e.g. air abrasion or selective infiltration etching [40]. A problem with silane promoted bonding is poor hydrolytic stability that leads to bond deterioration over time [48,49]. In this study, the specimens were stored in water for seven days prior to testing, and it is possible that with a longer water storage time, the bond would have started to deteriorate.

In the present study, the silane primer with MPS increased the bond strength of the ceramic brackets, whereas the universal primer with both MDP and MPS had no effect, even though it is suggested to bond to oxide ceramics [42,45–47]. Our findings differ from those of earlier studies that reported no effect of MPS on the bond strength of ceramic brackets [20], and an enhancing effect of MDP on bonding of ceramic brackets to ceramic substrates [50]. In addition to bonding with surface hydroxyl groups, another mechanism of action of silane coupling agents is based on improvement of surface wettability of the substrate by the monomers of the resin. This could explain the high bond strength of the samples in group cer2. It seems that the silane was able to improve the wettability of the ceramic bracket more than the universal primer.

Polycarbonate is a thermoplastic polymer, it is translucent and has somewhat higher mechanical properties than commonly used denture base polymer, poly(methyl methacrylate). Because polycarbonate is not a strong material, polycarbonate brackets are often reinforced with fillers or fibers. Polycarbonate brackets have been reported to yield lower bond strength values than metal brackets [19]. However, our findings indicate stronger bonding of the polycarbonate brackets compared to metal brackets.

Composite primers are primers which function either with the inorganic filler particles of resin composite or by acting with the polymer matrix by dissolution and polymerization. Typically composite primers are solvents and methacrylate monomers with photo initiators for polymerization [51,52]. Dissolution of the polymer substrate surface requires linear polymer structure of the substrate and therefore cross-linked polymers cannot be dissolved. Actual bonding is based on formation of interpenetrating polymer network to the interface of substrate and adhesive [53]. Composite primers can be mixtures of monomers and silanes, but the silanes have shown to be inactivated in the mixtures during the shelf-life time and the function of the silane component is questioned [54,55]. In the present study, the effect of composite primer or the adhesive resin on the bond strength of polycarbonate brackets was statistically insignificant. One way to significantly improve bond strength when bonding polycarbonate brackets with glass-fibers as fillers includes first exposing the fibers with sandblasting and then adding silane as a coupling agent [19].

The design of the bracket base is a key factor in creating mechanical retention, and it greatly affects the bonding properties of the brackets. The brackets in this study had very different types of base designs (Figure 1.), and each required a different debonding force. The more irregular the base of the bracket, the higher the surface roughness, which creates mechanical retention [56]. The small spheres of the ceramic brackets provide large surface area and undercut areas, which seem to provide better retention than the mesh on the metal bracket or the square protuberances on the polycarbonate bracket, even though the height difference of the base texture was lowest in the ceramic bracket (Figures 2–4).

In metal brackets, bonding at the bracket-adhesive interface is based on mechanical retention, and the macroscopically retentive design of the bracket base is therefore of primary importance [57–62]. In metal brackets with a mesh base design, larger mesh apertures have been shown to correlate with higher bond strengths, since it allows better resin penetration into the bracket base and allows air to be displaced from under the adhesive [63]. Improvement of the bond strength of metal brackets without enamel damage has been achieved by applying a metal primer containing 4-META (4-Methacryloxyethyl Trimellitate Anhydride) to the base of the bracket [64].

There are many different types of brackets available, and even within the same material category they differ in many of their properties, e.g. ceramic brackets include mono- and polycrystalline brackets, chemically bonding brackets and just mechanically bonding ones, and the size of the brackets and the design of the base vary considerably. Therefore the results of a certain type of a bracket cannot be generalized to comprise all the other brackets of the same material category, which adds difficulty to comparing the results of one study with another. However, with a growing body of research, the benefits and risks of different brackets will become clearer.

## Conclusions

- Bond strength values were highest for ceramic brackets, followed by polycarbonate brackets, and lowest for metal brackets.
- Silane primer increased bond strength when used with ceramic brackets, but otherwise primers had only minor effect on the bonding values.
- There is a risk for enamel damage with ceramic brackets when a silane primer is used and bond strength reaches very high values.
- Since the effects of the primers tested in this study were either insignificant or adverse, the use of these primers on the base of the brackets in orthodontic bonding cannot be recommended.
